# Referral Conundrum: Wait Times for Subspecialists Versus Comprehensive Otolaryngologists in the United States

**DOI:** 10.7759/cureus.91744

**Published:** 2025-09-06

**Authors:** Arman Saeedi, Tyler Muffly, Michaele Francesco Corbisiero, Gregory Burnet, Jingdian Huang, Brandon D Abell, Ethan Moore, Alireza Saatchi, Andy Ai, Blessed Asare, Lukas Maly, Drew C Gottman, Andrew J Tompkins, Cristina Cabrera-Muffly

**Affiliations:** 1 Department of Otolaryngology - Head and Neck Surgery, University of Colorado Anschutz Medical Campus, Aurora, USA; 2 Department of Obstetrics and Gynecology, Denver Health Medical Center and Hospital, Denver, USA; 3 Department of Otolaryngology - Head and Neck Surgery, Stanford University School of Medicine, Palo Alto, USA; 4 Department of Biology, University of Pittsburgh, Pittsburgh, USA; 5 Department of Otolaryngology - Head and Neck Surgery, Midwestern University Arizona College of Osteopathic Medicine, Glendale, USA; 6 Department of Biology, University of Colorado Colorado Springs, Colorado Springs, USA; 7 Department of Biology, University of Colorado Denver, Denver, USA; 8 Department of Biology, University of Colorado Boulder, Boulder, USA; 9 Department of Otolaryngology - Head and Neck Surgery, Ohio ENT and Allergy Physicians, Grove City, USA

**Keywords:** health services accessibility, otolaryngology, patient care, united states, wait times

## Abstract

Introduction: Timely access to care is a critical indicator of system efficiency and equity. This study aimed to investigate potential differences in new patient wait times for otolaryngology care between neurotology and pediatric subspecialists compared with generalists for matched-case vignettes.

Methods*: *A national cross-sectional audit study using a "mystery caller" approach was analyzed with linear mixed-effects regression Poisson models. A total of 682 physicians across 48 states, Puerto Rico, and the District of Columbia were included, representing three otolaryngology subspecialties. Mystery callers contacted otolaryngology physicians via telephone as patients with scripted clinical vignettes. Subspecialists were matched by city with generalists for the same corresponding clinical vignette. Callers requested the next available appointment. Wait times for new patient appointments were recorded and analyzed in R (R Development Core Team, Vienna, Austria) using generalized linear mixed-effects regression Poisson models.

Results: A total of 682 of 706 calls reached a representative (96.6%). The median physician age was 53 years. The median wait was 11.0 and 31.5 business days longer to see neurotologists or pediatric otolaryngologists (both p < 0.01) compared to generalists, respectively. Longer waiting times were also associated with physicians affiliated with universities (p < 0.001). Regional differences were also observed, with specific American Academy of Otolaryngology - Head and Neck Surgery (AAO-HNS) regions showing longer wait times. The pediatric and neurotology models both achieved conditional R-squared values of 0.97.

Conclusions: These findings indicate that subspecialty may significantly influence new appointment wait times, with pediatric otolaryngology patients experiencing the longest wait times. These differences in waiting times can have clinical and ethical implications for patient care, and future research should focus on optimizing care models to improve access to care.

## Introduction

Timely access to care is a critical indicator of system efficiency and equity. Shorter waiting periods before appointments correlate with higher patient satisfaction and perceived care quality [1]. Conversely, extended waits can jeopardize patient health and risk adverse outcomes [2]. These findings underscore the importance of access to care research focused on wait times (WT), particularly in specialized fields, such as otolaryngology - head and neck surgery.

This issue is especially relevant considering the broader implications of healthcare access disparities for vulnerable populations. Limited specialty care access can exacerbate health inequities [3], while efficient referral systems mitigate them [4]. Thus, timely access to specialist care, with appropriate referral patterns, is important for managing complex health conditions and improving patient outcomes [5]. Understanding and addressing disparities in access, especially related to referral bottlenecks, is essential to ensure equitable care.

In the United States, WT differences between Medicaid and privately insured patients have been documented, particularly within pediatric otolaryngology and neurotology [6]. Whether subspecialists generally face longer WTs than comprehensive otolaryngologists, however, remains unclear. Prior studies in otolaryngology have focused on insurance-based disparities, with little attention to intra-specialty access differences, which have been explored in general surgery [7]. With growing demand for otolaryngologic services, especially in regions with limited subspecialty access, it is increasingly important to understand how care is distributed across the specialty. This is increasingly relevant amid projected workforce shortages and national efforts to improve referral efficiency [8,9]. Identifying differences in WTs may reveal opportunities for coordinated care models, where comprehensive otolaryngologists manage overlapping conditions to reduce delays for patients. Delayed evaluation for routine issues may lead to prolonged symptoms and higher system costs [10]. Such models could help preserve subspecialist availability for complex cases, while improving overall system responsiveness [11]. In turn, this strategy may enhance continuity of care, particularly in underserved areas or locations distant from academic centers [12-14].

This study employs a national mystery caller approach to determine if pediatric otolaryngologists and neurotologists have longer appointment WT than comprehensive otolaryngologists for matched case vignettes [15]. Based on previous findings [6], we hypothesized that these subspecialists would have longer WT than generalists. Through matched clinical scenarios, this study identifies a potential systems-level variable related to access to care within otolaryngology.

## Materials and methods

Study participants

Comprehensive otolaryngologists, neurotologists, and pediatric otolaryngologists were selected to compare WT for matched case vignettes across otolaryngology subspecialties. Otolaryngologists were identified through systematic random sampling of entries from the American Academy of Otolaryngology - Head and Neck Surgery (AAO-HNS) patient-facing directory [16], with a second round of random supplementary searches used to ensure geographic representation and matched pair availability.

Identification of otolaryngologists

To achieve national representation, otolaryngologists were stratified by the AAO-HNS Board of Governors regions [17]. Duplicates were removed. Practices were geographically matched by city to reduce previously reported regional biases [6]. Calls were placed to individual named surgeons rather than to general practice lines. Subspecialist designation was determined using descriptions from the AAO-HNS directory and public-facing practice websites. Each call used standardized scripted clinical vignettes tailored to overlapping cases by subspecialty (pediatric otolaryngology vs. comprehensive otolaryngology and neurotology vs. comprehensive otolaryngology). Each vignette reflected standard, non-urgent diagnoses (Appendix A). Minimum sample size was calculated using a formula considering the target population, desired precision, and confidence level:

\( \textit{Minimum Necessary Sample Size} \;=\; \frac{N_{\text{population size}}}{1 + \bigl(N_{\text{population size}}\bigr)(\text{error margin})^{2}} \;=\; \frac{40000}{1 + 40000(0.05)^{2}} \)

Caller training

Eight callers were trained for consistent and reliable data collection. Calls occurred between 08:00 and 17:00 local time, excluding lunch (12:00-13:00), over one business week in June 2024 to minimize seasonal scheduling differences [18]. Calls to the same practice were at least 24 hours apart. No appointments were scheduled to minimize the burden on otolaryngology offices. To reduce potential bias, patient-identifying information was withheld [15]. Standardized scripts were used for all calls.

Interview procedure

Pilot calls were conducted one month prior to verify AAO-HNS directory numbers [19]. Callers, standardized to Blue Cross Blue Shield insurance, recorded the earliest available appointment dates for individual surgeons during data collection. Additional data included whether calls were unanswered, offices were not accepting new patients, or if visits with advanced practice providers (APP) were required. Calls on hold for more than five minutes were excluded; this threshold was chosen to both allow for the feasibility of making large numbers of calls and to reflect the busy lives of patients making calls during business hours. Each practice received two separate attempts before being considered unreachable. This standardized approach ensured consistent data collection across diverse practice types (e.g., academic vs. private practice) and reduced bias.

Informed consent

The University of Colorado Institutional Review Board (COMIRB 23-1303) deemed this study not human subjects research and did not require informed consent. As a courtesy after calls, debriefing letters were sent to participating practices.

Data collection

Data for each call were collected using REDCap, a secure web application for managing online surveys and databases [20]. Call data included the physician's office ID, call date and time, caller ID, appointment availability, earliest appointment date and time, if applicable, the reason for not eliciting a potential appointment time, and any additional notes. Surgeon demographics, practice type, and location were also recorded. Zip codes were converted to Rural Urban Community Area (RUCA; USDA, 2010 Census) codes; practices in metropolitan areas (codes 1-3) were deemed metropolitan, while others were considered rural. Characteristics were compared via chi-square analysis with Benjamini-Hochberg correction to identify post-hoc pairwise differences.

Data analysis

Analysis focused on comparing WT, in business days, between pediatric and comprehensive otolaryngologists, as well as between neurotologists and comprehensive otolaryngologists. Descriptive statistics were used to summarize central tendencies and distributions of WT, while inferential statistics were employed to determine the significance of observed differences. Data were analyzed using R (version 4.0.4; R Development Core Team, Vienna, Austria) [21]. Means, medians, and standard deviations were used to compare WT between groups. Additionally, mixed-effects linear regression analyses were performed to control for confounding variables, such as age, gender, Board of Governors region, hold time, having a call center or appointment line, number of transfers while on the phone, educational background of provider, rurality, and practice type. Visualizations, including dot plots, were generated to illustrate WT distributions and identify outliers.

## Results

We contacted otolaryngologists across 48 states, Puerto Rico, and the District of Columbia; only Maine and Wyoming were unrepresented due to exclusion during AAO-HNS regional random sampling. Of 706 total calls, 682 (97%) successfully connected with a representative. Among these, 97 (14%) did not accept new patients, 45 (7%) required referral, 15 required APP evaluation (2.2%), and 13 (1.9%) were excluded due to hold times exceeding five minutes. The final analytic sample included 682 otolaryngologists: 156 neurotologists, 197 pediatric otolaryngologists, and 329 generalists.

Table 1 summarizes provider demographics. The median age was 53 years (IQR: 44-61), and 553 (81.6%) were male. Neurotologists had the highest proportion of physicians over 60 (62, 39.7%), while generalists had the highest proportion under 40 (55, 16.7%). Male predominance was the highest in neurotology (137, 87.8%) and the lowest in pediatric otolaryngology (149, 75.6%). Generalists were most likely to hold a DO degree (16, 4.9%), compared to neurotologists (3, 1.9%) and pediatric otolaryngologists (2, 1.0%). Private practice was more common among generalists (243, 73.9%) than neurotologists (89, 57.1%) or pediatric otolaryngologists (84, 42.6%), while academic affiliation was most common in pediatric otolaryngology (113, 57.4%). All subgroup comparisons were statistically significant (adjusted p < 0.01), though no significant differences were found in practice setting, central appointment systems, call transfers, or AAO region distribution.

**Table 1:** Characteristics of surgeons called. ^a^Percentages are of the total available data points for each specialty. Four individuals had undetermined sex, and medical school location was undetermined for 70 general ENTs, 102 pediatric ENTs, and 29 neurotologists. For all values besides median age, data are presented as N (%). Chi-square test statistics are reported, and p < 0.05 is considered significant.

Variables	General ENT^a^ (N=329)	Pediatric ENT^a^ (N=197)	Neurotology^a^ (N=156)	Total (N=682)	p value	χ²
Median age, years (IQR)	51.0 (42.0, 61.0)	52.0 (44.0, 60.0)	54.5 (46.0, 62.0)	53.0 (44.0, 61.0)		
Age Category					<0.01	19.37
< 40 years old	55 (16.7%)	17 (8.6%)	12 (7.7%)	84 (12.3%)		
40-49 years old	90 (27.4%)	58 (29.4%)	37 (23.7%)	185 (27.1%)		
50-59 years old	87 (26.4%)	69 (35.0%)	45 (28.8%)	201 (29.5%)		
≥ 60 years old	97 (29.5%)	53 (26.9%)	62 (39.7%)	212 (31.1%)		
Sex					0.01	8.74
Male	267 (82.2%)	149 (75.6%)	137 (87.8%)	553 (81.6%)		
Female	58 (17.8%)	48 (24.4%)	19 (12.2%)	125 (18.4%)		
Medical School Location					0.37	2.01
US	223 (86.1%)	76 (80.0%)	106 (83.5%)	405 (84.2%)		
International	36 (13.9%)	19 (20.0%)	21 (16.5%)	76 (15.8%)		
Medical School Training					0.03	7.02
Allopathic training	313 (95.1%)	195 (99.0%)	153 (98.1%)	661 (96.9%)		
Osteopathic training	16 (4.9%)	2 (1.0%)	3 (1.9%)	21 (3.1%)		
Practice Setting					0.95	0.13
Metropolitan area	321 (97.6%)	193 (98.0%)	152 (97.4%)	666 (97.7%)		
Rural area	8 (2.4%)	4 (2.0%)	4 (2.6%)	16 (2.3%)		
Practice Type					<0.01	51.81
Private practice	243 (73.9%)	84 (42.6%)	89 (57.1%)	416 (61.0%)		
Academic	86 (26.1%)	113 (57.4%)	67 (42.9%)	266 (39.0%)		
Appointment Center					0.43	1.68
Yes	145 (44.1%)	96 (48.7%)	66 (42.3%)	375 (55.0%)		
No	184 (55.9%)	101 (51.3%)	90 (57.7%)	307 (45.0%)		
Number of Transfers					0.88	1.21
No transfers	302 (91.8%)	181 (91.9%)	142 (91.0%)	625 (91.6%)		
One Transfer	25 (7.6%)	15 (7.6%)	14 (9.0%)	54 (7.9%)		
Two transfers	2 (0.6%)	1 (0.5%)	0 (0%)	3 (0.4%)		
AAO Region					0.97	8.58
Region 1 (Connecticut, Massachusetts, New Hampshire, Rhode Island, Vermont)	14 (4.3%)	14 (7.1%)	10 (6.4%)	38 (5.6%)		
Region 2 (New Jersey, New York, Puerto Rico)	31 (9.4%)	23 (11.7%)	11 (7.1%)	65 (9.5%)		
Region 3 (Delaware, District of Columbia, Maryland, Pennsylvania, Virginia, West Virginia)	29 (8.8%)	16 (8.1%)	17 (10.9%)	62 (9.1%)		
Region 4 (Alabama, Florida, Georgia, Kentucky, Mississippi, North Carolina, South Carolina, Tennessee)	65 (19.8%)	33 (16.8%)	32 (20.5%)	130 (19.1%)		
Region 5 (Illinois, Indiana, Michigan, Minnesota, Ohio, Wisconsin)	51 (15.5%)	31 (15.7%)	25 (16.0%)	107 (15.7%)		
Region 6 (Arkansas, Louisiana, New Mexico, Oklahoma, Texas)	42 (12.8%)	24 (12.2%)	15 (9.6%)	81 (11.9%)		
Region 7 (Iowa, Kansas, Missouri, Nebraska)	20 (6.1%)	15 (7.6%)	9 (5.8%)	44 (6.5%)		
Region 8 (Colorado, Montana, North Dakota, South Dakota, Utah)	19 (5.8%)	8 (4.1%)	7 (4.5%)	34 (5.0%)		
Region 9 (Alaska, Oregon, Washington)	15 (4.6%)	7 (3.6%)	8 (5.1%)	30 (4.4%)		
Region 10 (Arizona, California, Hawaii, Nevada)	43 (13.1%)	26 (13.2%)	22 (14.1%)	91 (13.3%)		

The primary outcome was WT to new patient appointments. Overall median WT was 39 business days (IQR: 46). Pediatric otolaryngologists had a 31.5-day longer median WT (58.5, IQR: 56) and neurotologists had an 11-day longer median WT (40.0, IQR: 46) than their vignette-matched comprehensive otolaryngologists (27 and 29 days, respectively).

**Figure 1:**
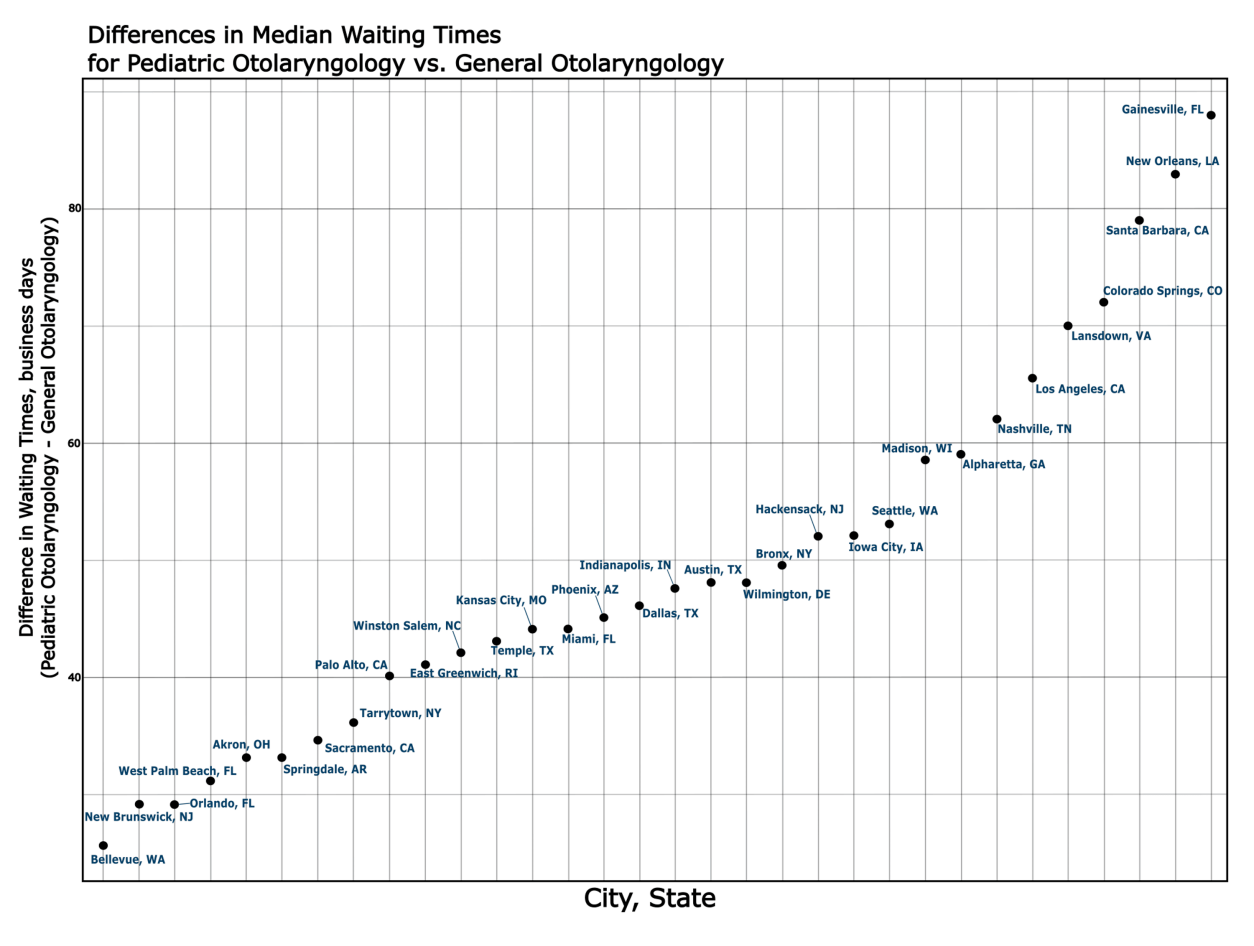
Scatter plot of the differences in median wait times (in days) for pediatric otolaryngology vs. comprehensive otolaryngology by city. This plot depicts cities where wait times (WT, business days) were greater for pediatric otolaryngologists.

**Figure 2:**
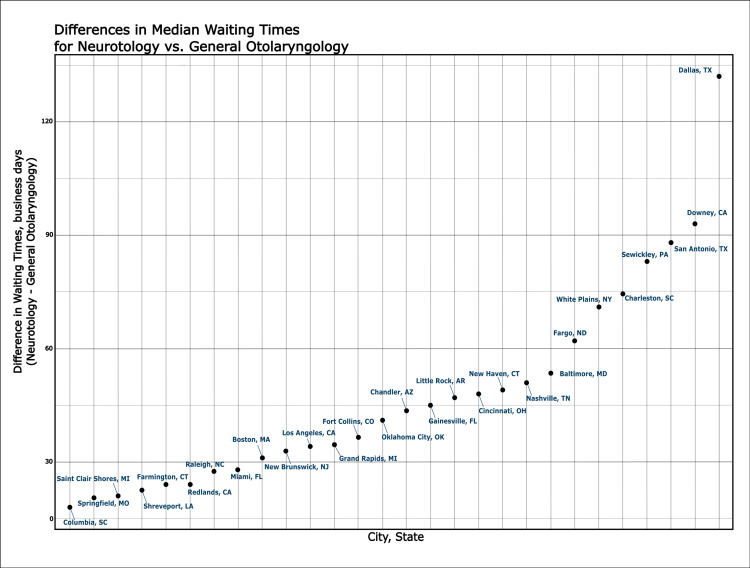
Scatter plot of the differences in median waiting times (in days) for neurotology vs. comprehensive otolaryngology by city. This plot depicts cities where wait times (WT, business days) was greater for neurotologists.

City-level variation is shown in Figures 1-2. For the pediatric vignette, the top 10 cities exhibited WT differences exceeding 50 days, with a maximum of 88 days. For neurotology, the top 10 cities had differences over 30 days, with a maximum delay of 117 days.

**Figure 3:**
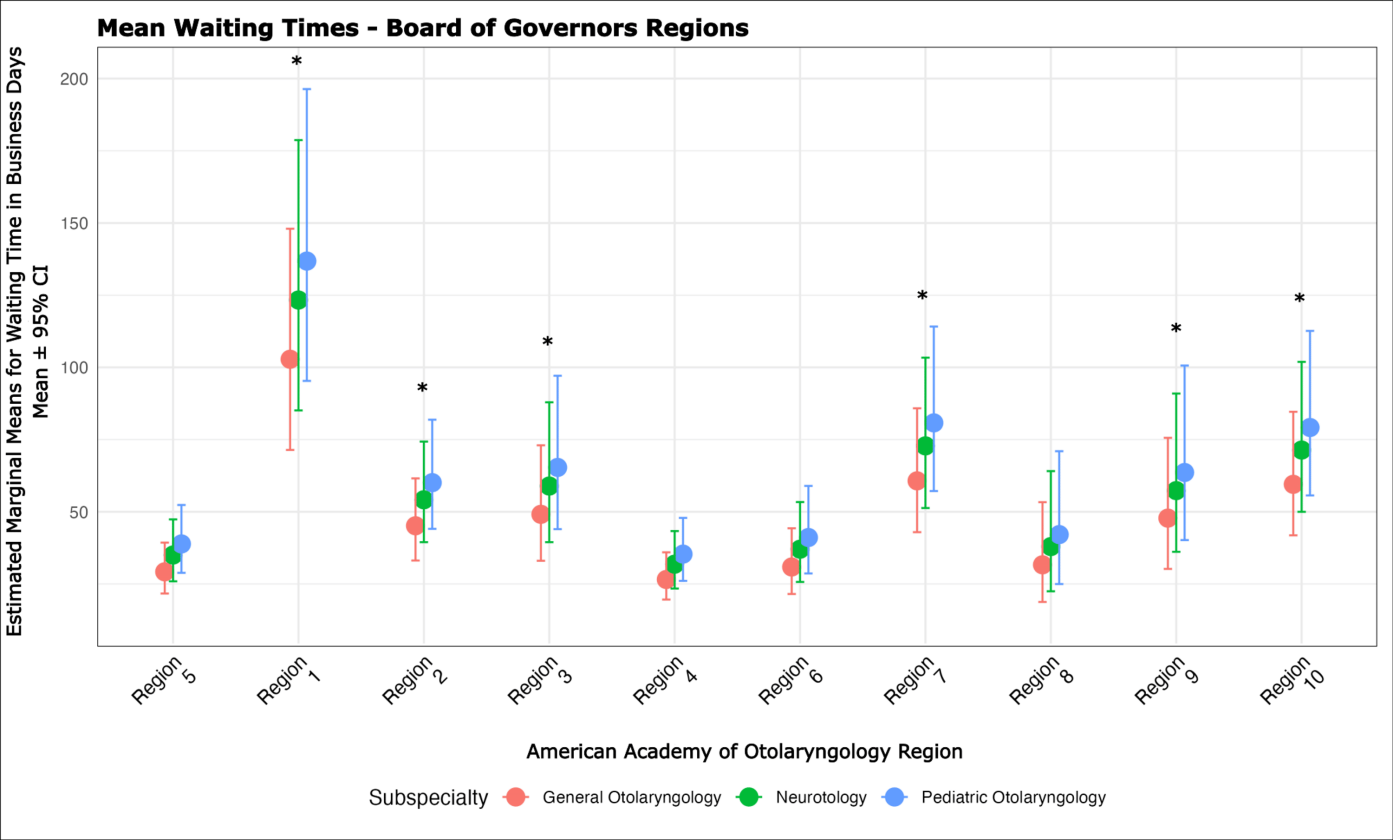
Dot plot of mean waiting times by AAO-HNS regions. This dot plot illustrates the mean waiting times in days across different AAO-HNS regions. Region 5, with the median overall waiting times, was chosen as the reference. It highlights that Region 1, which includes CT, ME, MA, NH, RI, and VT (incidence rate ratios (IRR): 3.52, z = 6.56, p < 0.001); Region 2, which includes NJ, NY, PR, and US Virgin Islands (IRR: 1.55, z = 4.59, p < 0.001); Region 3, which includes DE, DC, MD, PA, VA, WV (IRR: 1.68, z = 2.56, p = 0.011); Region 7, which includes IA, KS, MO, NE (IRR: 2.08, z = 4.03, p < 0.001); Region 9, which includes AK, OR, WA (IRR: 1.64, z = 2.10, p < 0.001); and Region 10, which includes AZ, CA, HI, NV (IRR: 2.04, z = 3.89, p < 0.001) had statistically significant longer wait times for new patients seeking care. Error bars indicate the 95% confidence intervals of the mean for each region. P < 0.05 in this generalized linear mixed model (Poisson) is considered significant.

**Figure 4:**
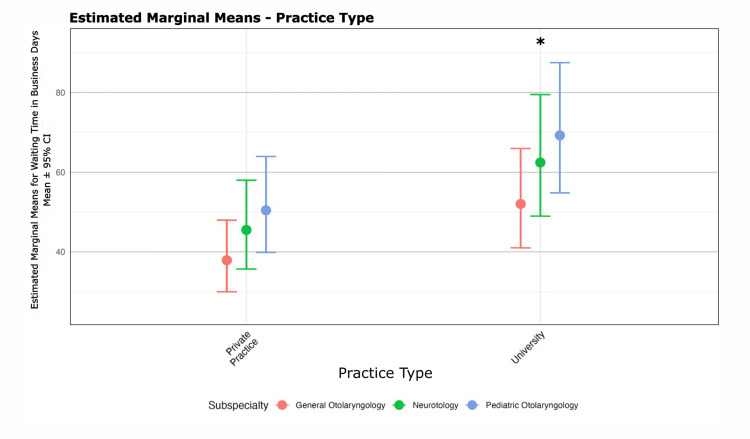
Dot plot of mean waiting times by practice type. This dot plot illustrates the mean waiting time in days across academic and private practice settings. This illustrates that academic practices (IRR: 1.37, z = 8.87, p < 0.001) had statistically greater wait times for new patients seeking care compared to private practice. Error bars indicate the 95% confidence intervals for each practice type. P < 0.05 in this generalized mixed linear model (Poisson) is considered significant.

Figures 3-4 display WT by region and practice type. AAO Regions 1-3, 7, 9, and 10 were associated with longer WT compared to the median region (Region 5). University-affiliated otolaryngologists had 37% (approximately 19 business days) longer WT than those in private practice.

**Figure 5:**
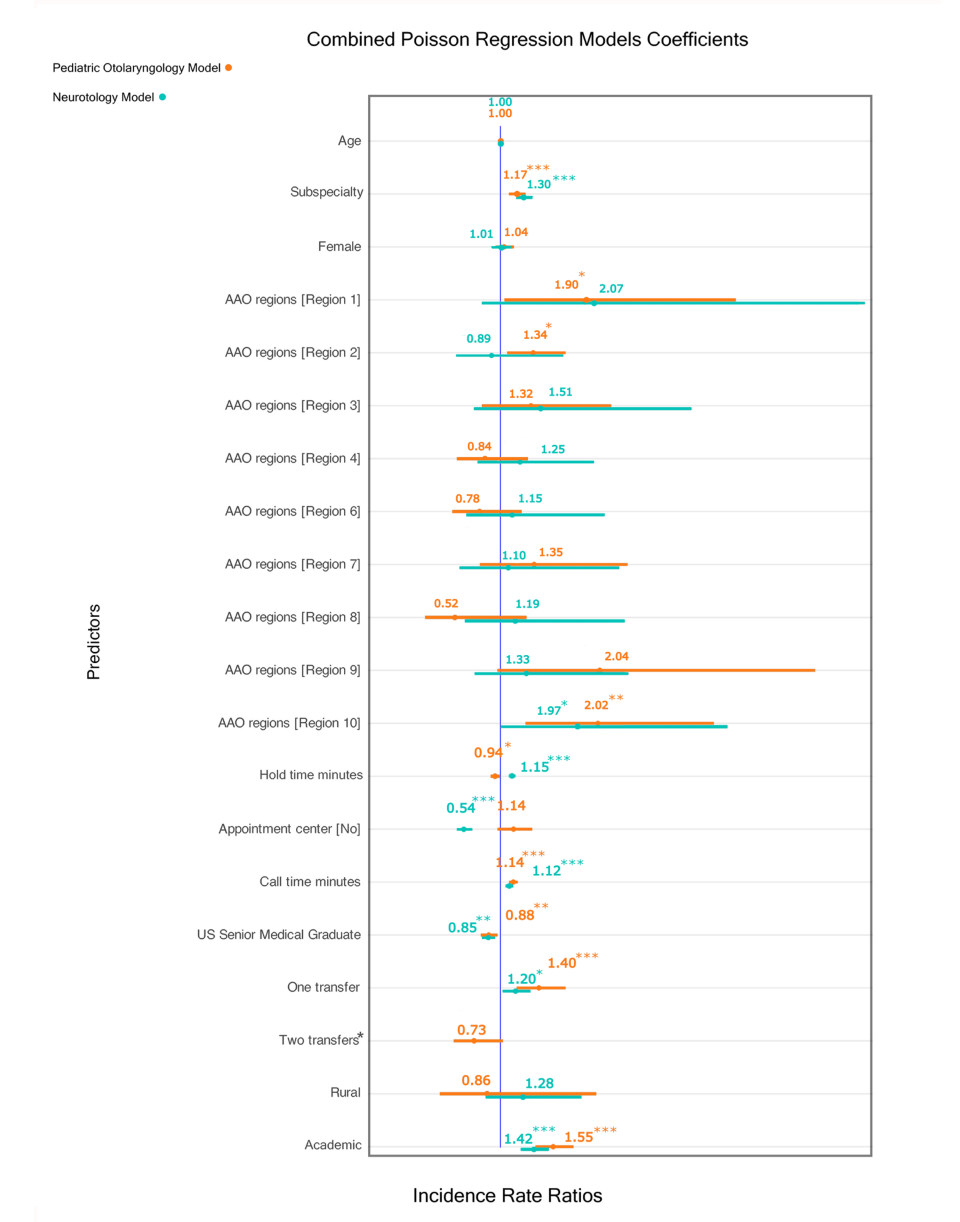
Forest plot of individual statistical models. This chart illustrates the coefficients that were found to significantly affect wait times. For both models, scheduling with fellowship-trained surgeons (pediatric IRR: 1.17, 95% CI: 1.09-1.26, z = 4.23, p < 0.001; neurotology IRR 1.30, 95% CI: 1.20-1.40, z = 6.50, p < 0.001), at academic sites (pediatric IRR: 1.55, 95% CI: 1.37-1.76, z = 6.80, p < 0.001; neurotology IRR: 1.42, 95% CI: 1.26-1.60, z = 5.68, p < 0.001), with longer call duration (pediatric IRR: 1.14, 95% CI: 1.09-1.18, z = 6.20, p < 0.001; neurotology IRR: 1.12, 95% CI: 1.07-1.17, z = 4.91, p < 0.001), and with one transfer (pediatric IRR: 1.40, 95% CI: 1.17-1.68, z = 3.67, p < 0.001; neurotology IRR: 1.20, 95% CI: 1.03-1.38, z = 2.40, p < 0.05) were significantly associated with longer wait times, respectively. No calls had two transfers in the neurotology vignette. P < 0.05 is considered significant.

Figure 5 presents results from Poisson mixed-effects models (GLMM). Both pediatric and neurotology models showed strong model fit (conditional R² = 0.97). Marginal R² values were 0.36 (pediatric) and 0.35 (neurotology). The random effect variance for city was higher in neurotology (0.610) than in pediatric otolaryngology (0.484), which may suggest greater location-based variability in neurotology access.

Subspecialist type and university affiliation were consistent predictors of longer WT. Pediatric and neurotology referrals were associated with 17% and 30% longer WT, respectively, compared to generalist referrals. US medical school graduates were associated with shorter WT in both models (12% shorter for pediatric, 15% shorter for neurotology; both p < 0.01). University affiliation was linked to longer WT in pediatric (IRR: 1.55; 95% CI: 1.37-1.76) and neurotology (IRR: 1.42; 95% CI: 1.26-1.60) models (both p < 0.001).

Regional effects varied between models. In the pediatric model, Regions 1 (IRR: 1.90, z = 2.10), 2 (IRR: 1.34, z = 2.55), and 10 (IRR: 2.41, z = 2.93) had significantly longer WT than Region 5 (all p < 0.05). In the neurotology model, Region 10 was significantly associated with longer WT (IRR: 1.97; z = 1.99, p = 0.046), and practices without central scheduling had shorter WT (IRR: 0.54; 95% CI: 0.45-0.65; z = -6.45, p < 0.001).

Model diagnostics supported predictive validity. Intraclass correlation coefficients were 0.95 (pediatric otolaryngology) and 0.96 (neurotology), which indicates strong within-group consistency. Root mean square error (RMSE) was 8.11 and 16.37, respectively, which reflects an average prediction error of ~8 and ~16 days.

## Discussion

Principal findings

Our nationwide study reveals significant disparities in WT for new patient appointments across otolaryngology subspecialties. Specifically, neurotology and pediatric otolaryngology appointments demonstrated markedly longer WTs, 30% and 17% longer, respectively, compared to comprehensive otolaryngology. These differentials persisted after controlling for potentially confounding variables, which indicates systemic rather than coincidental variation. University-affiliated surgeons had longer WT than private practitioners, corroborating previously published findings [6]. While surgeon age and sex distributions differed between specialties, neither factor was significantly associated with WT.

Geographic disparities in access compound subspecialty-specific delays [13,22]. Compared to the median appointment WT across all regions, Regions 1, 2, 3, 7, 9, and 10 had significantly longer WT. Stratified analyses revealed distinct regional patterns by subspecialty: pediatric otolaryngology had longer WT in Regions 1, 2, and 10, while neurotology demonstrated significantly longer WTs only in Region 10. These findings highlight the complex interplay between specialty-specific and geographic access barriers that collectively impede timely care.

Clinical implications

Protracted WTs for specialty care have direct and measurable consequences for patient outcomes. Care delays can impact patient health, quality of life, and healthcare costs [1,10,23]. Our findings indicate that, in numerous metropolitan areas in the US, patients in need of pediatric otolaryngology care may face delays of two to three months. These delays can significantly impact developmental milestones or result in preventable complications [24]. Additionally, pre-existing disparities in access to pediatric otolaryngologic care may exacerbate delays, and research suggests that WTs may be clinically significant for conditions such as otitis media with effusion, which are associated with speech delays, behavioral issues, and educational challenges [25,26].

Similarly consequential are the observed WT differences for neurotology. Dizziness, the chief complaint in our neurotology vignette, increases fall risk 12-fold in patients over 40 years old [27], and vestibular physiologic loss alone, with no identifiable cause other than aging, is estimated to cost a total lifetime societal burden of over $200 billion for the US population over 60 years old [28]. Although specific studies directly linking WTs to increased fall risk are limited, the general consensus in the literature supports the importance of timely intervention in vestibular disorders to prevent falls and improve quality of life. For patients with sudden sensorineural hearing loss, the AAO-HNS emphasizes the importance of early treatment [29]. Delays beyond eight weeks can drastically reduce the likelihood of hearing recovery [30].

These observed WT disparities highlight potential bottlenecks in the care continuum. Even in cases where nearby cities offer shorter WTs, travel-based solutions create new access barriers, particularly for vulnerable populations. The disproportionate impact of socioeconomic status and race on transportation access compounds these disparities, which highlights the complex interrelationship between clinical access bottlenecks and social determinants of health [31-33].

The paradox of proximity without accessibility is particularly interesting in our data. Although the average American resides approximately 20 miles from an otolaryngologist, with over 1,000 "high-access" coastal counties having a median travel distance of just three miles [34], our study demonstrates significantly longer appointment WTs in these same coastal regions. This suggests that geographic proximity to specialists does not necessarily translate to timely care access.

Alternative care delivery models offer promising solutions to these access challenges. Research in Canada, which experiences similar prolonged outpatient subspecialty care WTs, found otolaryngology eConsults reduced WTs nearly 29-fold compared to face-to-face consultations, with unnecessary in-person referrals avoided in 33.4% of cases [35]. With over 40% answered within 24 hours, eConsults provided rapid responses, and enhanced primary care-specialist communication and care efficiency [35].

Additional solutions to address these WT disparities are needed. Various interventions should be considered: (1) implementation of structured referral guidelines that direct appropriate cases to comprehensive otolaryngologists rather than subspecialists when clinically indicated; (2) development of regionalized care networks that balance patient loads across geographic areas; (3) adoption of dynamic scheduling systems that reduce no-show rates and optimize appointment utilization; and (4) integration of telehealth services, particularly for initial consultations and follow-up care, which has shown potential to reduce WTs by 30-45% [35]. Telehealth interventions offer particular promise for addressing both subspecialty and geographic disparities, as highlighted above.

Limitations

While our study revealed significant WT differences between subspecialists and comprehensive otolaryngologists, several limitations merit consideration.

To address challenges with data collection using the AAO-HNS public otolaryngologist directory, we conducted a pilot call phase, which revealed that the AAO-HNS directory contained errors, prompting a second search for surgeons to maintain statistical power, as highlighted in the methods section above. This search may introduce selection bias [36,37]. However, this mirrors real-world challenges patients face when identifying surgeons.

Surprisingly, scheduling in rural zip codes (2.3% of practices) did not significantly delay care compared to metropolitan areas. This observation may reflect a type II error, especially given the literature associating rurality with reduced access [38].

Additional limitations include our single-week data collection period in June 2024, which may not capture seasonal variations in appointment availability. Studies have shown that appointment scheduling can fluctuate by 15-25% throughout the year, with particular increases during winter respiratory infection seasons and summer months when elective procedures are more commonly scheduled [39,40].

Strengths

Despite these limitations, our study exhibits several notable strengths. It uses a large, nationally representative dataset with comprehensive geographic stratification, which enhances its generalizability across various otolaryngology practice settings.

This study reinforces the unique value of audit-style investigations, which have been underutilized in otolaryngology research. Collecting data through this “mystery shopper” approach, while matching generalist-specialist dyads by clinical vignette, strengthens findings by minimizing potential biases of appointment triaging based on illness.

Importantly, this study is among the first within otolaryngology to examine system-level differences in WT based on referral type, which compared subspecialist and generalist WT for overlapping cases. While previous studies primarily investigated patient characteristics, such as insurance status [6], this analysis provides real-world insight into how referral type impacts WT. These findings underscore the need to standardize and optimize referral patterns, which affect otolaryngologists managing these referrals. Previous work similarly identified sociodemographic and institutional factors influencing delays in otolaryngology care [41]. Together, these findings highlight opportunities to streamline and optimize the referral process to enhance patient access and care delivery.

## Conclusions

Otolaryngologists have fiduciary and ethical duties to promote the well-being of their patients, including facilitating timely care. Our study provides compelling evidence that patients may experience significant delays when scheduling appointments with pediatric otolaryngologists and neurotologists compared to comprehensive otolaryngologists for identical clinical conditions. Findings indicate that university-affiliated practices and regional variations further contribute to delays in care. These systemic disparities have critical clinical implications and may influence patient outcomes and increase healthcare costs.

To address these challenges effectively, the field must embrace innovative, patient-centered care delivery models. Optimizing referral systems and exploring appointment-level factors such as dynamic scheduling may enhance timely access to specialized care. Future research should focus on understanding the underlying causes of these differences and developing targeted interventions. These findings underscore the need for coordinated solutions that align specialty expertise with timely, equitable patient access.

## Appendices

Appendix A. Call vignettes by subspecialty

**Table 2:** Call vignettes by subspecialty.

Physician-Pair	Vignette
Comprehensive Otolaryngology/Pediatric Otolaryngology	The parent of a 3-year-old child with a history of recurrent ear infections and speech delay brings the child to their pediatrician. The pediatrician refers the child to a Pediatric / Comprehensive otolaryngologist for evaluation and potential placement of ear tubes to address the chronic ear infections and improve hearing and speech development. “Hello, I'd like to schedule an appointment for my 3-year-old daughter, who has a history of many ear infections and speech delay. Our pediatrician recommended an evaluation by a Pediatric/Comprehensive otolaryngologist to consider the placement of ear tubes. When is your next available appointment?”
Comprehensive Otolaryngology/Neurotology	A 55-year-old female patient presents to their primary care physician with a recent onset of dizziness, tinnitus, and hearing loss. The patient is referred to a Comprehensive otolaryngologist / Neurotologist for further evaluation and management. “Hello, I'm calling to schedule an appointment for my 55-year-old mother, who has recently experienced dizziness, hearing loss, and ear ringing. Her primary care physician has referred her to a Comprehensive otolaryngologist / Neurotologist. When is your next available appointment?”
